# The Ontology of Haag’s Local Quantum Physics

**DOI:** 10.3390/e26010033

**Published:** 2023-12-28

**Authors:** Gregg Jaeger

**Affiliations:** Quantum Communication and Measurement Laboratory, Department of Electrical and Computer Engineering and Division of Natural Science and Mathematics, Boston University, Boston, MA 02215, USA; jaeger@bu.edu

**Keywords:** local quantum theory, quantum field theory, interpretation of quantum theory, ontology, events

## Abstract

The ontology of Local Quantum Physics, Rudolf Haag’s framework for relativistic quantum theory, is reviewed and discussed. It is one of spatiotemporally localized events and unlocalized causal intermediaries, including the elementary particles, which come progressively into existence in accordance with a fundamental arrow of time. Haag’s conception of quantum theory is distinguished from others in which events are also central, especially those of Niels Bohr and John Wheeler, with which it has been compared.

## 1. Introduction

Rudolf Haag offered a framework for physics, Local Quantum Physics (LQP), as a theoretical conception broader in scope than a single theory and intended as a “strategic move” for the advancement of relativistic quantum theory [1] (it appears that the meaning of ‘framework’ Haag has in mind is similar to that surveyed and analyzed in [2], e.g., he mentions classical mechanics as understandable as such). The novelty of this framework is distinctly ontological: the entities it considers to be fundamental differ from those of standard quantum field theory (QFT); it demotes the field, giving the primary ontological role instead to physical events and their causes; it also considers space-time as secondary to these events, which are local in a primitive sense, and reifies their causes, which are unlocalized and link events into a network of elements of reality that grows as an evolving universe.

Haag’s foundational views were presented in a series of publications over two and a half decades, one of the first and the most known being an early interpretational sketch that appears as the final chapter in the second edition of his *Local Quantum Physics* (*LQP*), a book which lays out his mathematical treatment of relativistic quantum physics that had been developed over preceding decades through work with collaborators [3]. Reactions to the foundational aspects of LQP have been almost exclusively to that initial sketch, which garnered considerable attention; Haag’s more fully explicated worldview as fleshed out in later publications has gone largely overlooked, especially its ontological aspect (as Meinard Kuhlmann commented, “That Haag proposes an ontology for AQFT, namely an event ontology, has, as far as I can judge, not been greeted with great applause in the AQFT community, and, again as far as I can tell, not because of Haag’s specific conception but rather because of his very interest in philosophical, in particular ontological, considerations” [4]. This work has been overshadowed by the significance of the theorem bearing his name that is understood to indicate inconsistencies in the so-called interaction picture method of formalizing quantum dynamics; for a representative example discussion, see [5]). For this reason, a thorough review and brief remarks regarding Haag’s full ontological position are given here, along with a discussion of these reactions that indicates its differences from the foundational views of others, such as Niels Bohr and John Wheeler, who also emphasized the importance of events in physics.

The picture of the physical world of LQP differs from traditional ones in which physics is assumed to take place among a collection of ontologically fundamental fields and/or particles within a self-subsistent space-time arena. And, although both the LQP framework and Wheeler’s Meaning Circuit model of the universe, inspired by Bohr’s notion of phenomenon, portray the physical instead primarily via sets of actualized events, with space-time and other physical entities seen as secondary to them (cf. [6]), the events of LQP are unambiguously objective, causally connected, and realized in accordance with the forward march of time: There is no place in it for any “circuit” requiring observer participation for their coming into existence. LQP aimed to overcome difficulties arising from previous approaches to quantum theory, especially those related to space-time localization, and Haag’s foundational elaboration of it denied any role for observation or consciousness in physics. And, again unlike Wheeler (and Eugene Wigner), Haag consistently rejects all reference to consciousness in physics, holding strictly to the view that “events are not tied to observation” ([7], p. 66); these objective events are absolutely fundamental.

In contrast, especially to Wheeler, Haag gave no role to the information obtained by observation. He avoids the thought-experimentation pioneered by Bohr and continued by Wheeler and Wigner, as well [8]. These differences are significant in that there is a long history of criticism of the investigation of the foundations of quantum theory among physicists in response to the prominence of the thought-experimental method, the oftentimes relative lack of mathematization, occasional reference to the consciousness of observers, and recent suggestions that information plays a primary role in physics (for a discussion of the critique of the observation and measurement-based approach to physical events as made forcefully by John S. Bell, see [9]).

The ontology of Haag’s physical world is one of objective, localized interaction events involving unlocalized particles and other objects. These events are understood to support space-time, although he views demonstrating this as an ongoing endeavor, and the successive causal realization of events is understood by him to correspond to a fundamental direction of time (Kuhlmann argues that there is an apparent divergence of Haag’s view from that of mainstream AQFT in that “it seems that [Haag’s] event-ontological conception is incompatible with the very approach of AQFT where space-time regions are primary, and not derived from an event structure via ordering relations” [4]. About this, see Section 6, below). “Ultimately one might say that the significance of the notion of space-time reduces to the description of causal relations between events.” ([7], p. 68) After a brief summary of the methodological and mathematical background of Local Quantum Physics in Section 2, its ontology as characterized by Haag is explicated in Section 3. The differences of Haag’s LQP from other approaches to quantum theory are described in Section 4 in light of the initial reactions to *LQP*. Haag’s conception of the universe as an unfolding causal network of events is sketched in Section 5.

## 2. Approaching the World via LQP

A primary goal of the LQP framework is the determination of the ontological structure of the physical world. “Every scientific endeavor begins by dividing complex situations into individual elements” ([10], p. 23). Haag considers this traditional methodology, *on the whole*, to have been appropriate but ultimately limited: “Of course the holistic criticism of reductionism…is justified. But…[we] have to divide [nature] and, as it turns out, we are highly successful in doing so up to some point” ([10], p. 23). In LQP, this is carried out by considering equivalence classes of events, times, spatial regions, and objects all forming ensembles in the general sense, not specifically those of probability theory (cf. [11], Section 2), in order to capture the regular behavior of the world. The formation of these classes is viewed as necessary for enabling well-justified physical statements on the basis of inductive inference.

Haag conceives the ontology of LQP as an extended, objective realm composed of events and their causal connections, about which reliable knowledge can be obtained: “I believe that a theory based on the notion of real events can be developed in a way which is not at variance with established experimental results” ([10], p. 26). In LQP, *events* are the most elementary entities, which become causally connected, for example, when they appear as interactions involving stable elementary particles and are indicated by the traces they leave [12]. Much like Wheeler, Haag views the history of physics as naturally setting the stage for his own approach to nature through the failures of previous approaches. But, whereas for Wheeler, the failure has been primarily one of the dependence on physical law, Haag points to the weaknesses in methodology and ontology of the mechanical approach to physics, which LQP is intended to rectify.

“The first is the division into individual objects, beginning from bulk material and proceeding with its subdivision into molecules, atoms and ultimately ‘elementary particles’. The reduction of physics to the study of interaction between such objects is the point of view of mechanics”.

([10], p. 24)

The determination of the structure of quantum entities such as atoms, which were not well understood before quantum theory, was only partially successful via the notion of the quantum system in the development of *quantum mechanics* (QM), the second step of this history. “Quantum mechanics takes over this [mechanical] vocabulary but shows that the notion of individual objects can have a precise meaning only under very special circumstances.” ([10], p. 24)

One of the valid requirements for physics that Haag sees in mechanics is *physical isolation* of the sort found in stable objects. “It is one of the triumphs of Quantum Mechanics to achieve a classification of stable objects and to describe their distinctive attributes.” ([10], p. 24) However, the implementation of even the early forms of quantum theory demonstrated the limitations of the independent-systems approach to quantum physics due to the way this very requirement is implemented there:

“…isolation can never be perfectly realized. It is an asymptotic notion. Furthermore it does not even suffice to allow us to speak of an individual object. There may be entanglement with other far separated objects”.

([10], p. 24)

Indeed, it is well known that interacting objects are readily entangled; cf., e.g., [13], Section 1.5. Recognizing the concomitant approximate character of the ontological reductions achieved in the quantum realm is central to Haag’s view, and, for this reason, he prefers to refer to the ontological *model* of LQP. The realm of QM is one of systems with finite numbers of degrees of freedom, but the fundamental entities of QFT, quantum fields, possess an infinite number of degrees of freedom. Quantum fields are, therefore, not entities of the same sort as those of QM.

The third step in this progression of physics is the approach of quantum field theory incorporating the principle of relativity. But, although the field has been a useful mathematical tool for the implementation of the locality principle, in LQP, more complex structures, namely, nets N of algebras of observables are understood to ground relativistic physics. A difficulty in the standard approach to QFT that concerns Haag is that having a physically determinable field value at a space-time point would involve an unlimited amount of energy, and point-like field interactions have been widely viewed as giving rise to divergences in calculations in traditional QFT. Although this requirement might appear operationalistic, it is understood in the LQP framework as one on the environments in which events can occur with physically acceptable values. To keep such values finite, LQP considers values only on open, bounded regions O instead, and nets N on local such regions are given priority over fields; it is the mapping from space-time regions to algebras of such local observables that is physically significant in this approach, which is outlined below. More broadly, LQP considers events localized in (Minkowski) space-time M, but with the corresponding space-time regions *assigned to quantum events rather than vice-versa* and the space-time metric independent of physical states. (The relation of events to space-time location is further discussed here in the following section).

LQP is even further removed from the mechanical approaches than is traditional QFT: Not even quantum fields appear in the fundamental ontology of LQP; rather, they are connected to it secondarily. “In fact, the relation to quantum fields is not as close as originally believed. In particular, it is important that it can also incorporate extended objects which generalize the field concept.” ([14], p. 2) Nor does the mathematical approach of LQP involve quantizing a classical field, which would involve the assumption that the basic dynamical variables underlying the operators of the quantum state space are fields. In LQP, fields serve merely to provide a coordinatization (of the pertinent net of operator algebras), in the sense that a mapping from space-time coordinates to field values is available. For Haag, “The naive idea that a field ϕ assigns to each space–time point *x* an operator ϕ(x) in H is not tenable…” ([14], p. 4). Rather, “One may consider a field as an ‘operator valued distribution’ on a suitably defined domain in H or as a sesquilinear form on this domain…The theory is completely described by a finite number of covariant fields (each having a finite number of components).” ([14], p. 4).

In some regards, the mathematics of the approach proceeds along the lines of what is now called Axiomatic Quantum Field Theory, which was pioneered by Wheeler’s student Arthur Wightman and others, but with the important difference that, as just noted, quantum fields do not appear at a fundamental level, and space-time is not assumed to be an a priori given arena in which interactions simply take place. Also, the Wightman axioms do not guarantee the existence of a net of local algebras of bounded operators, and such a net does not guarantee the existence of a field system satisfying the Wightman axioms, either ([1], p. 107). Broadly speaking, the relation of LQP to Wightman’s axiomatic description of quantum fields and vacuum expectation values (VEVs) is, from Haag’s point of view, analogous to the relation between coordinate-free description and a coordinate description of geometry (cf. [15], p. 4). (H.-J. Borchers later showed that the quantum fields form local equivalence classes the members of which describe the same particles, particle scattering, and localized algebras; [15], p. 4. A summary of the essential structure of the LQP can be found at [1], pp. 141–142).

Haag offers the framework of LQP as a scheme with a “degree of flexibility,” something he sees in classical mechanics as well, when it is considered independently of the specification of a set of forces. Despite the approximations involved in providing its model of a real, objective world, it was, in his opinion, almost inconceivable that any experimental result in high-energy physics could be incompatible with the LQP framework. (According to Haag, in the future, only a change needed to make quantum theory general relativistic could be envisioned.) In the ontology of LQP, a continuously changing entity causally linking events is classified as an *object*, and an abruptly and briefly present entity is classified as an *event*. Haag takes “the notion of ‘event’ as a primary concept” ([7], p. 66), and

“the relation to space-time is provided by the events. Each event marks roughly a region in space-time, the sharpness of which depends on the nature of the event. No independent localization properties of [causal] links is assumed.”

([12], p. 736)

These links are formalizable as directed causal “arrows.” Thus, the distinction between the two sorts of real entity in LQP is intimately connected with the relationship of quantum theory and space-time, as detailed here in Section 5, where the conception of the events of LQP in relation to physical processes is discussed in some detail.

A particle is understood by reference to a previous (“source”) event and *can produce a signal in a single detector but never in two or more spatial distinct ones at any single time*, where a ‘detector’ is a general sort of environment in which an event can arise. The formal setting for LQP is provided under the assumption that objects appear that facilitate detection-like (in the most general sense) responses, interpreted as events each confined to an open region of M of finite extent, represented by an element of an involutive (*-)algebra, from which the representatives of general observables can be constructed, their compatibility corresponding to commutativity, which is assumed to hold for space-like separations; cf. [16]. Haag calls the LQP approach *algebraic* because it characterizes a theory by a net of algebras of local observables ([3], p.VII), as detailed below, and for a proper theoretical structure within it, one needs:

“(i) to endow the set of possible events and possible arrows

with further mathematical structure,

(ii) to state the restrictions in connecting events by arrows,

(iii) to give the scheme by which the probability for the appearance

of a specific pattern (subset of events and arrows) may be computed

from information about the past history,

(iv) to establish the relation to an observer and his instruments,

essentially to classical space-time” ([17], p. 248), which, for Haag,

is a greater theoretical project.

The relationship of events to space-time, the common-sense ontology of the laboratory, and possible knowledge of data, i.e., need (iv) is accomplished, broadly speaking, as follows. Space-time is considered representable by the four-dimensional Minkowski space M possessing a causal and metric structure, but only as “a derived concept” where the “notion of event replaces the marked point in space-time, the notion of causal ties between events corresponds to the ordering relations in Leibniz’s understanding of the role of space”, and “the unity of time and space is essential in this picture” ([16], p. 139). (Haag recognizes the anti-Leibnizian character of this but justifies it by noting agreement with Bertrand Russell that “the technique of mathematical physics continued to be Newtonian…When we deny absolute space, while continuing to use what we call points in mathematical physics, our procedure is only justified if there is a structural definition of point and of particular points in the theory.” ([16], p. 138). “In a more ambitious analysis one might hope to use the geometry of patterns as a substitute for space-time” ([12], p. 736)). With (i)–(iv) in place, it is possible to “compare [this] picture to quantum field theory in the regime of low density and conspicuous events. In this regime causal ties correspond to particles and events to collision processes between particles." (ibid.) An event “will be very diffuse” for low-energy events and “rather sharp in the case of high energy-momentum transfer” ([10], p. 29). A relatively detailed such comparison is given in Section 2 of [17] and discussed further here in Section 5.

The event-and-object ontology accords with the formalism of the framework of LQP, which Haag characterizes as a “fusion of the (classical) principle of locality at the level of special relativity with the concepts and the mathematical structure of quantum physics” ([16], p. 135), as now follows. The set of bounded operators B(H) acting in quantum Hilbert space H is considered together with regions O of M. A quantum theory incorporating special relativity is considered as given via a map from the set K (an orthocomplemented lattice with ordinary set theoretic union and intersection) of the causally complete regions in M, into the set of von Neumann subalgebras of B(H). (Given a subset S of B(H), its *complement* considered as the commutant $S^{\prime }$ is a von Neumann algebra, and $S^{\prime \prime }$ is called the *causal completion* of $S^{\prime }$; the set N of von Neumann algebras an orthocomplemented lattice.) In particular, a map is introduced from the causal completion of the open regions into the collection {R(K)} in the set of von Neumann subalgebras of B(H)

K∈K⟶R(K)∈N,

that respects the lattice operations of K and N, where N is called a “net” of local algebras and is taken to be such that:

(i) There is a unitary representation of the group of translations *a*,

i.e., a⟶U(a)∈B(H) (with a positive energy requirement on the

spectrum of the generators $P_{\mu }$ of translation);

(ii) There is a translation-invariant ground state (the vacuum) Ω vector

satisfying $P_{\mu }\Omega =0$; and

(iii) All R(K) are isomorphic as von Neumann algebras to the (unique)

hyperfinite factor of Type III. ([16], p. 136)

Haag argues that these requirements are strong and suffice as a basis for constructing specific theories *within* the framework of LQP, that is, one comparable in reach to those of conventional quantum field theory: “the net of algebras defines the theory, including the full physical interpretation”; “Once the net is given, we can analyze its physical predictions in terms of particles, collision cross sections, etc.” ([14], p. 8) (The general outline of how this is to be carried out is provided in some detail in [17], pp. 248–250.) It is not surprising to note, then, that Haag’s quantum causality (Haag duality) differs from Einstein causality as imposed in QFT via constraints on field operators [15]. Indeed, three different causality-related requirements can be considered within LQP, namely:

$$\begin{array}{c} \text{Einstein causality}\;\;\;A\left(O^{\prime }\right)\subseteq A{\left(O\right)}^{\prime };\\ \text{Causal completion}\;\;\;A\left(O\right)\subseteq A\left(O^{\prime \prime }\right);\\ \text{Haag duality}\;\;\;\;\;\;\;A\left(O^{\prime }\right)=A{\left(O\right)}^{\prime }. \end{array}$$

Haag duality is a special case of Einstein causality, and causal completion is closely related to it; cf., e.g., [15]. (The *causal completion* is the casual complement of the causal complement of a region O, i.e., $O^{\prime \prime }$, where the *causal complement* is the set of all points which are space-like related to all of the points of O of M, and $O\subseteq O^{\prime \prime }$; in the case of equality, one has the maximal extension consistent with Einstein causality.) LQP imposes time-like causality as a quantum version of hyperbolic wave propagation. (The Haag duality condition and its significance is discussed in some detail by Haag’s collaborator Bert Schroer in [15], pp. 4–7).

## 3. The Entities of Local Quantum Physics

The relationship of the long-recognized physical entities of QM and QFT, such as elementary particles and atoms, to the primary elements of the LQP ontology is as “ ‘causal ties’ (or ‘links’)” to those events from previous events. And, for example, in an experiment, there is, metaphorically speaking, a “‘decision’ involved in the realization of a particular result in a measurement. Dirac called it ‘a decision by nature’…we might better say: it is a decision in nature instead of by nature” ([10], p. 25) because future events are not precisely predetermined. (The detailed relation to time is discussed below in Section 6). The corresponding facts are knowable, in principle, if the supporting data are, fortuitously, conserved into the future via later causal relations between events.

In LQP, the events are more or less sharply localized (to space-time regions), but the objects serving as causal links between them are *not*.

“This corresponds to the orthodox statement that ‘a particle does not have a position at any given time unless it is measured’. In our words: a causal link does not have space-time attributes (apart from those due to the events it connects)…A link is a messenger connecting a source event with a target event. In the simplest case (low density situation) links correspond to the aforementioned objects (stable particles)”.

([10], p. 28)

And, in the time between events, such “a causal link, say a particle, represents a potentiality until it has fulfilled its mission i.e. until some target event is concluded…its ‘state’ is a propensity assignment relevant for the occur[re]nce of a subsequent event.” ([10], p. 29) (Haag comments on his use of propensity with the following: “Although it is somewhat tedious to keep using the unfamiliar term propensity instead of probability I decided to make this effort whenever speaking of not yet realized possibilities in individual cases. It is clear that a propensity cannot be verified.” ([7], p. 63). For a discussion of the notion of potentiality, cf., e.g., [18]). With the realization of such an event, any links to it are reified. Thus,

“we can speak of the individual electron which was ejected from a metal surface by a radiation pulse and subsequently caused a click in a detector. But it makes no sense to talk about an individual electron without reference to specific events”,

whether they have come to be known or not ([10], p. 28). “The interaction of the particle with the detector produces an event and this is localized in space-time. Prior to this event we cannot assume any localization of the particle because there exist interference effects…”. ([19], p. 222) Here, the *detector* is a general notion, which indicates a portion of the environment and subsumes (at least parts of) purpose-built detection apparatus, but is not restricted to them.

Despite its lack of specific properties, space-time localization, or space-time trajectories, there is, nonetheless, a specific sort of *unity* to an object such as a particle that can be considered an individual, a “staying together” in the sense mentioned above: Given several detectors, the object is incapable of causing the excitement of more than one ‘detector’ at a time, being in that limited sense “permanently singly localized"; Haag means by this that such an individual cannot cause any time-coincident events, and points out that in the case of elementary particles, this is “equivalent to the well known requirement that it is a state with sharp mass value” ([19], p. 222). He explains this in relation to one of the most important examples in the history of quantum theory.

“Under what circumstances can we regard the ionization of an atom by a photon as a closed event?…The formalism of present day Quantum Theory suggests that there is no sharp boundary. While the definition of individual objects is limited by entanglement the definition of individual events is limited by coherence.”

([10], p. 26)

Both entanglement and coherence “have their root in the assumed unrestricted validity of the superposition principle” governing quantum probability amplitudes ([10], p. 26), and “the probability of a single event, considering only its backward causal ties, is not a meaningful concept. One must consider probabilities of patterns and these will not, in general, factorize.” ([17], p. 250).

This presents a challenge to the idea that a “theory could be regarded as an extrapolation from the realm of objective appearance and could ultimately yield an [exact] ontological model of the universe” ([11], p. 78) such as prevailed in mechanics. Haag’s approach is neither positivist nor direct-scientific realist, but rather, he advances a modest realism, and views the LQP framework, ontologically speaking, as having “a limited range of applicability. I do not believe in the possibility of a ‘theory of everything’ achieving a mental isomorphy with the world of appearances.” ([10], p. 26) As Haag sees it, in physics, “a theory aims at providing an ontological model which should be in harmony with known experimental facts but is not synonymous with them” ([10], p. 26), and he rejects the idea of a classical–physical type of ontology for quantum systems as given via some local hidden-variable model due to the nonseparability of quantum states, as evidenced in the violation of Bell-type inequalities, despite his use of the term “element of reality” commonly associated with Einstein, Podolsky, and Rosen and their argument for the incompleteness of the quantum state description. “The surprising behavior of ‘atomic objects’ shows that the ‘elements of reality’ cannot be those in which classical-type physics believed.” ([20], p. 309).

Haag argues that theorizing in physics should be carried out more subtly, according to “an ‘as if’ realism” in which physics by its very method does not transcend the dividing line between an assumed real outside world and the world of the mind with its impressions, emotions,…”, and he precludes any essential role for consciousness of one who might be in command of measurement apparatus as well. For the conception of events as measured, “What is important is that we are forced to use a realistic language...which amounts, in the last resort, to a description of the placement of material bodies in space-time.” ([7], p. 61) (However, this “placement” is given by events in LQP, not by reference to a *background* space-time, but one corresponding to their placement in a causally ordered network of quantum elements of reality as discussed in Section 6, below.) Haag’s view is that elements of traditional physical language persist in quantum mechanics because, in it, one assumes it possible to consider a particle, say, the electron as a ‘physical system’, which is convenient. But difficulties arise if it is assumed that such a system has all of the classically attributed properties.

Haag’s position lies between the two sides of the Bohr–Einstein debate in regard to the appearance of values in measurements. For example, considering the measurement involving an electron he argues that “If we look without bias at the experiments alluded to, we must recognize that the ‘element of reality’ in the position measurement is a dot on a photographic plate or a flash from a scintillation screen” ([20], p. 309), and “These are indeed not properties of the electron but properties of the interaction process of the electron with another system. They are ‘events’ characterized by a reasonably sharp position in space and *in time*” ([20], p. 309). Such quantum processes are related to space-time locations as follows. Quantum processes can be understood in relation to the basic quantum fields, which associate to each open region O in space-time an algebra A(O) of operators on Hilbert space, the algebra generated by all smeared out fields Φ(f), where *f* is a test function having support in O. The elements of A(O) represent physical transformations that might take place there. The net N of algebras provides a mathematical basis for the physical characterization of processes associated with events. The quantum fields provide a coordinatization of this net of operator algebras, in that a mapping from space-time coordinates to field values is available. As example events, Haag discusses “well known high energy experiments where we see by inspection in the individual case the existence of a collision center from which the tracks of particles emerge” as well as complementary situations where the momenta of incoming matter are extremely precise and localization can be thought of as specified by the interaction length; cf. [12], Section 3. (Haag points to work of David Finkelstein [21] as one method of viewing the structure of space-time based on the consideration of a set of events considered prior to it. As noted above, the relation of quantum events to space-time itself is considered to be part of a much larger, ongoing research project. Another possible approach to making this relationship precise is that of causal sets; cf. [22]).

In analyzing events at small scales, there remains the question of the nature of causes, such as elementary particles. For example, “What about the electron itself? We experience it as a causal tie or link between two events, its ‘birth’ in the electron source and its death (or transmutation) in the interaction with the detector.” ([20], p. 309) By contrast with elementary particles, Haag supposes that it is possible to consider macroscopic objects such as detection apparatus to have space-time locations. “We regard macroscopic bodies as ‘real objects’ and statements about their placement in space and time as ‘real attributes’. The word ‘real’ just means here that such objects, events and their space-time attributes belong to common experience shared by many persons and do not depend on the state of consciousness of an individual.” ([23], p. 14) But, unlike macroscopic objects, the completed causal links, such as elementary particles, between distant localizable events are not localizable in space-time. And they can give rise to non-classically correlated events as well, particularly among atomic-scale objects:

“The formalism of Quantum Theory suggests that most (if not all) of the ways by which we try to subdivide the universe must be regarded as approximations whose worth depends on particular circumstances. This applies most strongly to the division into individual objects”.

([11], p. 78)

Ultimately, “the best division we have, the one which constitutes the rock on which present theory is built, is the division of space and time” ([11], p. 78). The focus on observables in quantum theory which has predominated, he suggests, “may veil the central point that all measurements in atomic physics ultimately end by the detection of an event with its localization in space and time”, within physics itself ([19], p. 225); “…we have emphasized the need to regard the notion of ‘event’ as a fundamental, primary concept, ultimately *replacing* the concept of observable. It establishes the bridge to reality and space-time.” [emphasis mine] ([19], p. 232). This differentiates Haag’s approach from the “orthodox Copenhagen” approach to quantum theory, which is based on notion and mathematics of the observable.

## 4. Reactions to Haag’s Foundational Views

Expanding on the content of the first edition of *LQP* four years later, Haag offered a second edition, which contained a new chapter presenting his basic foundational views: “Principles and Lessons of Quantum Physics. A Review of Interpretations, Mathematical Formalism, and Perspectives.” The critiques of Haag’s LQP from those working in the foundations of physics appear to have been exclusively of *Local Quantum Physics* itself in these editions. The most detailed foundational reaction to LQP appears in a review of the second edition by Nicolaas Landsman, who sees Haag’s interpretation as a combination of sympathy for and criticism of the “orthodox Copenhagen spirit.” (This review [24] was published in the journal *Studies in the History and Philosophy of Modern Physics* the same year.) Landsman claims that Haag “stresses the need for the ‘Heisenberg cut’ between a system and an observer, and quotes Bohr in saying that the observer plus instruments side is described by classical physics”, together with occasional signs of “an operationalistic attitude: physical relevance seems equated with measurement and observation, and even special relativity is motivated by the need for a convention for the synchronization of clocks.” These cursory comments exemplify the reaction to Haag’s initial expression of foundational views, that is, those of the early-to-mid 1990s.

However, the mention of Bohr and the quote in question serve as an historical point of reference to what had been a standard approach to quantum physics, which, for Haag, serves as a background for the explication of his own notion of quantum event, and it cannot be rightly said that, for him, physical relevance is equivalent to measurement or observation, at least not of the sort found in the Copenhagen school, with its observables, or that his views accord with the views of those who later took a human subject as essential to the occurrence of events. Haag is highly critical of “the special role of the observer” in “the standard formulation” associated with the latter group. “In the interpretation advocated by some eminent scientists, especially by London and Bauer, von Neumann, Wigner, the ‘observer’ means ultimately the realm of consciousness, a realm beyond the range of physical arguments.” ([7], p. 64) Most eminently, Wheeler, who spent time at Copenhagen as a postdoctoral researcher, placed the observer centrally within reality in his Meaning Circuit. Haag was greatly opposed to this extreme version of the “orthodox” interpretation having roots in Copenhagen, calling it “unhelpful” ([7], p. 65). And he sees the use of the word ‘classical’ to describe practical physical language in experimentation as an unfortunate distraction ([7], p. 61). In LQP, instead, “The ‘language of classical physics’ stressed so much by Bohr as indispensable for the observer (to enable him to tell what was done and learned) …may be reduced to the description of geometric relations in the placement of various macroscopic bodies and the coarse events observed in space and time.” ([23], p. 10). Such placement is not inherently classical–physical, and in LQP, space-time is not an arena of physical activity given a priori.

Although Bohr himself did not advocate an active physical role for consciousness, his emphasis on giving a role to observation and measurement when founding the “orthodox Copenhagen spirit” introduced a new element into the foundation of physics which others, inspired by his views, took further. Haag comments that “The standard language places the observer in a central position. According to common language an observer is a human being; at least he has to be equipped with a mind.” ([17], p. 246). In a later piece, Haag explicitly precludes any essential role for observation in the occurrence of events: “In order to rule out clearly the mental interpretation I propose to generalize the term ‘result of observation’ to the term ‘event’, a concept not tied to any passage into consciousness or to the performance of an experiment” ([10], p. 26). Haag is only in good agreement with Bohr and conservative followers in regard to the importance of intersubjective agreement for *knowledge* given objectively appearing measurement data, rather than in the granting of any role to observation per se in physics; he does not recognize either a metaphysically significant ‘observer side’ or classical descriptions as acceptable in fundamental physics, endorsing only the pragmatic consideration of macroscopic objects as located in space-time.

Like Landsman, Meinard Kuhlmann sees LQP as having an operationalist aspect, arguing that Haag had offered “a muddle of operationalism (and thereby anti-realism) on the one side and then, surprisingly, ontological thought’s [sic] about AQFT on the other side…” [4] (Note also that much of the content of Haag’s writings on foundational questions for LQP discussed here in previous sections appeared after critique, and, unfortunately almost all criticism, even that appearing much after *LQP* itself, addresses the book alone, and overlooks or ignores these later papers.) However, Haag’s interpretation of LQP is not genuinely operationalist—it is a realist one founded on the specifically quantum sort of causation, namely, that discussed here in the previous section; he seeks “operational principles” as a way of motivating a mathematical formalism that captures its causally connected ontology, but as unneeded for the interpretation of LQP, and points out that some of the fundamental elements of quantum theory either have no “simple operational meaning” or are “not operationally justified”; cf. [1], pp. 6, 37.

Although Haag does use the terms ‘operational’ and ‘detector’, like his mention of the Copenhagen approaches, these are also essentially points of reference for the indication of the sort of environment in which there can be a probabilistically caused event under the requirement that energy within any real situation be finite; although a detection-like environment is required for an event to arise, it is not required of such ‘detection’ that it arise due to an agent acting in a specific manner or via purpose-designed ‘detector’; the mathematics involved captures an interaction, whether or not a recognized form of detector could be designed to interact accordingly. And it is indicative that Haag considers the question that would likely arise in a genuine operationalist treatment, “ ‘How can we construct a measuring apparatus for a given self-adjoint operator?’ ”, to be an “often voiced pseudo-problem” ([19], p. 225).

“Experimental equipment is part of objective appearance, irrespective of whether it is wil[l]fully constructed and placed somewhere. The concept of an ‘observable’ involving the choice by an experimenter to provide the scene for the emergence of facts is a deus ex machina with serious flaws”.

([11], p. 80)

As noted here several times above, events occur of natural causal accord, independently of whether there is any control or specific method involved. Haag does mention what he regards as an ‘operational definition’ of ‘particle’ in a publication predating *LQP*, as follows:

“[In] a battery of detectors the particle can excite at most one detector at any given time. A particle, though it has no sharp position, is ‘permanently singly localized’, i.e. it cannot produce any two-or-more fold greater equal time coincidences. This constitutes an operational definition of the concept ‘particle’”.

([19], p. 222)

But that remark, like the mention of Bohr’s perspective and previous interpretational ‘orthodoxy’ serves only to explicate LQP via reference to experimental practice and standard theoretical approaches in the foundations of physics as understood by physicists; again, use of the term ‘detector’ here is meant in a broad and abstract sense, that of an environment enabling an event; it does not imply an adherence to *philosophical* operationalism. Indeed, Haag explicitly describes his view as a form of realism, as shown here in the previous section. The significant characteristic of an individual particle, for Haag, is its *causal unity*, not the method by which it is measured.

Landsman notes generally that, in regard to foundational issues, “most modern authors treat measurements as particular kinds of interactions, and equally, many are keen to abandon the so-called eigenvector–eigenvalue link (which Haag implicitly does in his discussion of particle localization)”, as well as that “the idea of a relational definition of factualization is common to most versions of the many-worlds interpretation and the modal interpretation.” ([24]) This sort of approach roughly characterizes what Jeffrey Bub calls the New Orthodoxy, seen by some as succeeding the approaches of the predominant Copenhagen ‘spirit’ [25]. But Haag’s approach to factuality, with its “decisions in nature,” is not genuinely relational; Wigner’s friend does not appear in LQP. Nor is his approach a thoroughgoing modal one, even though he does use the word ‘propensity’ to indicate quantum probability as quantifying possibility; see Section 6, below, for a discussion of this. It does not conform to this “new orthodoxy.” Landsman remarks that “Combining all these [elements] with the insistence on space-time localization seems typical of Haag, however. Much remains to be elaborated, but the seeds for an attractive realist interpretation of quantum field theory appear to be there.” ([24]) According to this assessment, at the time, Haag needed primarily to provide a fuller interpretation of LQP by the lights of the study of the foundations of physics and to distinguish clearly his views from those of the “Copenhagen spirit.” That Haag did later do, as shown further below. Landsman’s review ends with the comment, “Despite these somewhat critical remarks…for those interested in the foundations of quantum field theory Haag’s book is second to none. Indeed, everyone interested in modern physics should read it.” [24].

In *LQP*, Haag differentiates his foundational approach from some of those in the ‘Copenhagen spirit’ (the ‘old orthodoxy’) by, among other things, fully excluding consciousness from any role in bringing about physical events. (He thereby addresses any concern about a cut-via-consciousness. There has been, at best, a muted reaction to these clarifications, which is one of the motivations of the current paper.) Considering the language of the latter, he says, “Let us start with the word ‘observable.’ It suggests that there is an observer. Does this have to be a human being? Certainly in the discussions of the early days of Quantum Mechanics no other interpretation was intended.” ([12], p. 733) Indeed, “In the orthodox language of Quantum Mechanics the observer occupies a central position and the only ‘real events’ are the measuring results [but] this narrow view is not forced upon us by the lessons of Quantum Physics” ([12], p. 733). And he argues that

“one can hardly doubt that facts similar to measuring results occur in nature irrespective of whether they arise in a planned experiment and enter the consciousness of an observer. …Thus we may assume that a measuring result is an event whose reality status is no better than that of other events in nature…”

([12], p. 734)

Nor does the LQP approach to measurement take classical physics as fundamental, but still recognizes that space-time location happens to be useful for specifying experimental circumstances. To Haag, such location is an appropriate and efficient description of the placement of apparatus in the laboratory that benefits experimenters or observers of natural events more generally in the practical acquisition of knowledge of objectively present data vis-à-vis space-time: “The asymmetric treatment of the emergence of facts as an interaction between a (large) measuring instrument with a (possibly small) system is pragmatically useful but not appropriate in a fundamental theory” ([11], p. 80). Thus, one sees that Haag relates the LQP approach and previous approaches to foundational questions in quantum theory for clarity, rather than adopting them in an eclectic “muddle.”.

After the discussion in *LQP*, Haag makes it clear in further publications that, in his view, “It is the independence of the physically relevant phenomena from the state of mind of any individual human observer which is meant if we speak of an ‘exterior (physical) world’ called nature” that is the subject of physics ([10], p. 25), and that he considers the relationship between mental content and the external world provided by the methods of experimental practice as adequate. Haag’s realism imposes requirements for the objectivity of data, while recognizing the importance of intersubjective agreement about observations in the provision of knowledge: “The essential criterion for accepting an element of consciousness as the *cognition* of a counterpart in reality is the consensus between many observers…If this is satisfied, the agreement of all people concerned is adequate for treating the said observation ‘as if’ it were an element of an outside world.” [emphasis mine] ([19], p. 221). It appears, then, that this is what he means in *LQP* when stating that “With regard to the interpretation there is no basic disagreement with the epistemological analysis of Bohr but an appeal to accept that physical theory always transcends the realm of experience, introducing concepts which can never be directly verified by experience though they must be compatible with it.” ([20], p. 322).

Kuhlmann also commented supportingly, at least, of the formalism developed: “In the most general ontological perspective…[Algebraic]QFT should be the leading object of study, supplemented by investigations about standard QFT.” ([4], p. 58) (And, he also noted “AQFT, even if this was not initially intended, has a great deal to say about the most general nature of the ultimate ‘building blocks’.” [4], p. 58.) AQFT is an approach in which Haag was a pioneer, although he thought the name inappropriate; LQP is not a field theory per se. A third perspective on LQP, in particular, in the first (1992) edition of *LQP*, is provided by Gordon Fleming, who largely concurs with Landsman’s summarial assessment, pointing out—in a 2002 review of Paul Teller’s book on QFT—that, in Haag’s presentation, there was indeed a framework rather than a fully worked out theory [26]): “…in some instances, additional theorems, as yet unproved, would need to be established to mathematically justify [transition amplitude] calculations or, in other instances, the calculations, ’though rigorously doable, are dauntingly complex when so executed” [26] (Fleming continues, “Of course the book in question is aimed at the precise formulation of QFT and its structure. Applications are not the primary concern…We also learn that to the date of its publication no non-trivial examples of interacting QFTs in 4-dimensional Minkowski space-time had been rigorously demonstrated to be internally consistent. Not one. [Footnote:] I have recently been informed that one such model has now been proven consistent. I have not yet tracked down the technical details to assess the significance of the example.” [26].).

The goal in the final, interpretational chapter of *LQP* is to present LQP as a “conceptual picture which…incorporates in a natural way essential lessons of Quantum Physics but also raises new questions” ([10], p. 23). Although it is arguably a sort of AQFT, Haag views LQP as an “alternative language, closer to the intuitive picture of the working physicist in many areas” that “is not only possible but warranted. It needs, however, a different conceptual picture ultimately implying also a different mathematical structure.” ([12], p. 733). After the appearance of *LQP*, Haag continued to elaborate upon the basic ontological model of LQP in relation to these and other foundational concerns, while, indeed, leaving additional mathematical work to be accomplished and further detail to be provided in order to arrive at a specific theory in the usual sense, again, because LQP is intended as a broad framework from within which more specific theories could be developed. (Note that, with the exception of the final short commentary chapter [20] and his consideration of space-time in [16], only publications subsequent to *LQP* are considered here). Some of the needed work was mapped out by Haag after *LQP* and has been pursued by others since; cf. [15].

## 5. Events, Causes, and Space-Time in LQP

The greatest physical shift in the move to LQP regards the relationship between quantum events and space-time. Haag notes that, in previous physics, it has been the case that “(useful) theories start from space-time as its primary concept. It provides the arena for physics, or, in another picture, the vessel into which matter is put and events take place.” ([16], p. 138). The word *event* had the sense of a marked point in space-time as the most elementary concept of relativity theory, but the space-time locations of events in LQP are not connected by world lines composed of such marked points ([17], p. 247). The primary concept of LQP is the objective *local event*, and physical events and the causal relations between them conceptually precede space-time: “It is the event, the interaction process, to which the attribute of localization can be assigned” rather than to self-subsistent individual objects interacting ([10], p. 29), and

“if we take a boundary in the evolving category of events and ties then there is no set of alternatives for a ‘next’ event. Many subsequent patterns are possible and a set of alternatives (needed for the concept of probability) results only when all ties emanating from the boundary events become saturated”.

([17], p. 248)

There is, instead, “the aspect of potentiality i.e., the set of all possible patterns. It is not likely that they can be embedded in a 4-dimensional continuum but the set of possibilities itself may be regarded as replacing the aforementioned arena.” ([10], p. 30).

In LQP, there is a causal order involving these ‘ties’ (or ‘links’) between earlier and later events, which also become ‘elements of reality’ upon the appearance of the later events:

“A causal link also has attributes which we can regard as real. They are structural properties. In the case of the electron they are for us today only its mass, electric charge, magnitude of spin, and magnetic moment. For the next generation of high energy physicists they may include some internal structure”.

([20], p. 310)

Such “a link becomes established (an ‘element of reality’, if one wishes) only when the target event is concluded. In general the source event does not even determine the nature of the potential links originating from it. Potential links belong to the realm of possibilities, not facts. This illustrates the sense in which we can speak of individual objects.” ([10], p. 24) Unlike for the events, no independent localization property can be assigned to causal links ([10], p. 29).

The causal links of LQP, which include the elementary particles as characterized by the quantities identified by Wigner’s symmetry-based treatment [27], are genuine elements of physical ontology, along with the events they cause, and can be indirectly associated with the space-time locations of those events. In LQP, it is possible only under special circumstances to proceed from these ontological elements “to the materially defined systems of quantum mechanics, claiming for instance that in some large region of space-time we have precisely an electron and a proton whose ties to the rest of the world may be neglected” ([20], p. 298). One motivation for the formal approach of LQP, outlined here in Section 2, is that “one cannot expect to know the assignment of a specific [Hilbert-space] operator to a given [realistically available particle] detector…”, but one can “describe the set of all detectors indicating an event in a specific [open] space-time region” indicated by a net of algebras including all *environments* acting in a causal trace realizing fashion ([19], p. 232). (The formal elements are of the approach “often, but inappropriately, called ‘Algebraic Quantum Field Theory’, because we feel that it provides the simplest and most natural formulation in which the relevant principles can be expressed and it also provides a powerful mathematical structure which can be precisely described and applies to a wide area.” [14]. Algebraic Quantum Field theory originates in von Neumann’s operator algebras. And Axiomatic Quantum Field Theory, pioneered by Wightman, originates in the theory of operator-valued distributions and progressed toward the use of rigged Hilbert spaces; cf. [28].).

A requirement for a material object to have an individual, independent (temporary) existence is *isolation*, i.e., “the requirement that in a large neighborhood we have a vacuum-like situation.” This is possible for objects when “originally close together, become widely separated, still remaining isolated. Then we might want to consider them as separate individuals and wish to assign to each of them some notion of ‘state’ (some attribute)” ([20], p. 299). (The description of particle histories relevant to these considerations can be found in [1], pp. 288–289.) This, of course, can only be understood in LQP in terms of the separation of the *events* they could give rise to under the constraints imposed by time-like causality. This would be verifiable by approximative extrapolation from what is seen in cases of continual monitoring, such as in a bubble chamber when there is a significant mean free ‘path,’ but it is generally impossible to consider even apparently isolated systems as so due to the persistence of correlations such as those violating a Bell-type inequality, as is often required for conservation of a common property after a close encounter or interaction. This image of the world is far removed from any classical view, for example, Maxwell’s view of a universe of atoms wherein “the foundation stones of the material universe remain unbroken and unworn. They continue this day as they were created—perfect in number and measure and weight” (J. C. Maxwell, as quoted in [29], p. 82; [3], p. 298), including relativistic versions of classical mechanics.

In addition to the challenge of rigorously founding space-time on the causal network of events, the viability of Haag’s approach in *LQP*, which is taken to be entirely general and understood via algebras of observables, has been questioned in regard to situations such as quark confinement because particle clouds are considered to surround charged particles; cf. [24]. Nonetheless, as Landsman notes, for Haag, “The physical meaning of localization is that elements of [the algebra of observables] are ‘observables which can be measured in the region $O^{\prime }$ (p. 110). However,…particle detectors can only be localized up to infinite tails.…Haag would presumably say that: ‘we do not want to bother and we need not know in such detail’ ” [24]. Haag later argues specifically that “it seems highly unlikely that we can attribute to high energy events a localization of [, say, intrinsic size of $10^{-15}$ cm] though we have no means of verifying this in the individual case. Thus the indirect check by means of dispersion relations could be explained by the existence of sharply localized events rather than sharply localized observables.” ([12], p. 742).

Difficulties arising from the consideration of observables in quantum mechanics are argued by Haag to be better addressed by reference to sharply localized events. These events are readily idealized, for example, “a star of tracks in a photographic emulsion or bubble chamber emerge more and more conspicuously. Such a phenomenon has a center, the apex of the star, the dominant event in our sense. Its position in space-time is sharp as compared to the range of locations allowed by the preparation of the beam.” ([17], p. 247). And “This hinging of basic concepts to asymptotic situations which are only approximately realizable emphasizes the need for idealizations. Idealizations cannot be avoided if we want to talk about any subdivision of the universe though this does not necessarily have to be done in terms of particles and collisions.” ([12], p. 735) Haag discusses such situations specifically. For example, he considers the situation in “the reaction area of a storage ring high intensity high energy beams of electrons and positrons”, the results of possible collision processes, and quark-antiquark production resulting in hadronic jets which are registered in arrays of monitored detectors, and argues from these that “For many purposes it suffices to understand by the term ‘event’ just a detector signal. But …one can push this to finer distinctions: Invisible processes like microscopic triggering events or elementary reactions…which are reconstructed from many secondary detector signals.” ([19], p. 233). Haag argues that this is not problematic for the physicality of the picture of LQP and the objective reality of the past—as they would appear to be for Wheeler’s Meaning Circuit model, for example—because the existence of past events is in no way dependent on any mental observation: Some events correspond to the operation of designed and monitored detectors, while others happen ‘in the wild,’ leaving traces that go unnoticed due, for example, to a lack of amplification of space-time distance from scientists.

Even after the appearance of humanity, all but very few events in the universe are ever directly observed, of course. Others are conceivable but are unrealizable. “The simplest type of event is a collision process between particles, well isolated from other matter and closed by the spatial separation of the reaction products. Its mental decomposition into subevents or ‘virtual events’ (as in Quantum Field Theory) is a useful model but no individual existence of the virtual events can be claimed.” ([12], p. 735) The irreducible representations of the Poincaré group indicate the states of a stable system [27]. “This simple system is a single particle. Its attributes are a value of the mass and the spin, which define a group character” ([23], p. 9). This helps explain why collisions among such particles are important for physics:

“The reason why the simplest systems play such an important role for observations is due to the circumstance that in many experiments the partial state pertaining to a large but limited region of space-time can be very closely approximated by the restriction of a global single particle state to the region”

([23], p. 9)

it is also possible to characterize “simple global states such as vacuum, single particle states, thermodynamic equilibrium states to specified temperature and an array of chemical potentials”, and the division of space-time “allows us to define ‘local observables’, (roughly) representing detectors and coincidence arrangements of detectors”, etc. [23], p. 9).

The limitations of the above approach to probing the world are well recognized by Haag. As shown above, he explains how the conception of fundamental processes, for example, particle scattering, inevitably involves extrapolating to events in LQP. In a situation such as high-energy interactions, “The events in which we are interested are not the detector clicks. They constitute only a minor disturbance of the processes.” ([11], p. 80). Instead, there is “a localization of the vertices of significant events which is much sharper than the overlap of wave functions, the extension of a detector and presumably much sharper [than] the reconstruction from the traces reveals.” ([11], p. 80) So, one cannot consider “such an event as the measurement of some local observable. The refinement of the notion of event is a question of extrapolation (determining the limitations in the extension of objective appearance).” ([11], p. 80).

## 6. The Evolving Universe of LQP

According to LQP, the temporal evolution of the universe is connected directly to the time-directed probabilistic appearance of events. (He notes that this point in on that had been pointed out for many years by Carl Friedrich von Weizsäcker, citing as example the latter’s paper [30].) “The content of objective appearance depends on time, or rather it increases with increasing time because we can find records of past phenomena but cannot see the future.” [11]. The real universe becomes greater with the continued causality-constrained appearance of these events. But any quantum states attributed are not to be given any direct ontological interpretation.

“We distinguish sharply between facts and possibilities as it is demanded if we believe in intrinsic indeterminism. …a ‘state’ subsumes the probability assignment for the realization of a pattern of future events. It is not an element of reality.…This implies, however, that the arrow of time must have fundamental significance. A fact is created; it did not exist prior to some time and it is irrevocable. Thus the picture describes an evolving universe with a growing pattern of events representing the respective past as opposed to an open future. The pattern of realized events determines the probability assignment for subsequent growth of the pattern (the state)”.

([10], p. 28)

That is, only the past events, causal links, and current events (and the networks of them) are real; the future is only *potentially* real. Events are continually being realized probabilistically in the present and give rise to more elements of reality that are intermediary causal links from past, realized events, and hence also to the structure of space-time. The arrow of time corresponds to the contingency of the events.

The boundary between the realized elements of the physical world and what is merely possible in the future *advances with the present*. “The universe is regarded as an evolving history of facts; mathematically as an evolving graph or category whose points are the events and whose ‘arrows’ are the causal links. Its shifting boundary separates a past which is factual from a future which is open.” ([7], p. 66) It is also unsharp due to the limitations on the localization of events:

“the boundary (the present) remains unsharp since we have no clear definition of elementary events, no sharp subdivision of coarse phenomena. Of course the ‘arrow’ does not refer to clock-time but to the ordering of causal relations between events…” ([11], p. 82)

It thus corresponds to “a cut between past and future relative to some group of conservers. On the one side lies the extended objective appearance of the past, considered as factual. On the other probability assignments for future patterns of phenomena which are infer[r]ed from past facts.” ([11], p. 82) But this boundary is not the ‘Heisenberg cut’ and, as shown above, does not involve consciousness or, even, the brain of a conscious observer per se. The “conservers” mentioned here are not minds but portions of the universe itself which can serve as indicators of past events that can be traced in the future due to the continued forward-directed networking of causal links; a past event may be discovered to have taken place by anyone who happens upon another event (in the future of the first event) that it actively gave rise to (in that person’s present).

From the perspective of LQP, the directionality of time can be viewed more formally as follows.

“The events are partially ordered. Event β is called *later* than α if it is the target of a sequence of [causal] arrows of which the first has its source in α…The boundary is formed by all events which are sources of unsaturated arrows, arrows which have not (yet) found a target. The structure is evolving by the formation of new events absorbing unsaturated arrows so that events which were on the boundary now become inner”. ([17], p. 248)

And there is no inconsistency between this fundamental directionality of time and the time-reversal symmetry of quantum theory: “The significance of the arrow of time is encoded in the existing theory by the spectrum condition for the energy-momentum of states entering in the characterization of detectors (which provide one contribution for the probability of an event).” ([23], pp. 14–15). (Haag also emphasizes that the time reversal operator leaves the sign of the energy unchanged.).

Thus, the evolution of the universe envisioned, which is based on physical deeds of nature that arise independently of consciousness, also differs strongly from that described by Wheeler’s Meaning Circuit model and its Participatory Universe (about which, cf. [6]). In contrast to Wheeler’s conception of cosmological history, in particular, there is no suggestion of reaching into the past to actively participate in the creation of past events from the present via observer participation, even though each unlocalized causal link becomes real upon the appearance of the (probabilistically) caused event with which its linking ends. “The event can be assumed to exist whether we observe it or not and obviously only few events will be documented. The sharpness of the mentioned space-time position depends, of course, on the nature of the event” ([20], p. 2) as discussed in Section 5 above.

Haag also distinguishes his position from that of Bohr by the manner in which the irreversibility of processes arises.

“Bohr mentions the ‘essential irreversibility inherent in the very concept of observation’ but attributes this to the needed amplification and thus implicitly to the realm of statistical mechanics. Here we replaced the concept of observation by the concept of event and, while amplification is needed for *recognition*, it is not considered as an essential prerequisite for an event”. [emphasis mine] ([7], p. 66)

In LQP, it is isolation of a quantum process that is necessary for the realization of events:

“…in the presently available theory the notion of event or fact indeed needs some idealization but…this does not mean complex systems or macroscopic amplification but rather *isolation in a sufficiently large space-time volume*.” [emphasis mine] ([20], p. 311)

Such isolation is present in low-density situations, where “a collision process between particles (elementary or composite), leading from an initial configuration to a final configuration and fixing some approximate position in space-time, may be regarded as a closed event, a fact.…” [20] in the manner described here in the previous section.

In practice, regarding knowledge of events and their causes, one gathering knowledge must be (i) “in surroundings in which material bodies are concentrated to occupy a small part of available space with ‘lots of vacuum’ around”; and (ii) it must be “that we can guarantee stability in the repeated use of the same equipment in several experiments. Without these lucky circumstances our ability of acquiring individual knowledge would be in poor shape” ([7], p. 61). Thus, scientific knowledge depends on objective events and is subject to the limitations on their discovery.

## 7. Conclusions

Rudolf Haag offered an approach to relativistic quantum theory with his Local Quantum Physics framework, through which he expected that the known results of quantum field theory would be surpassed. The formalism of LQP was presented in unified form in his book *Local Quantum Physics*. After its appearance, there was recognition of the need for a fuller interpretation of LQP, which Haag subsequently provided in the book’s second edition and in a number of later publications. His initial foundational discussion included its relationship to past physical theory and these preceding interpretations, from which his interpretation differs fundamentally. There were several criticisms of both editions of *LQP*, most of which were in reaction to this mention, though not the active use, of the approaches within the Copenhagen school and of operational definitions, with which, some argued, Haag’s views have an ambiguous relationship. After these critiques, until the end of his life, twenty years after the appearance of the second edition of *LQP*, Haag endeavored to clarify his interpretational position by revisiting fundamental questions and clarifying its ontology, and he clearly distinguished it from that of previous physics.

With regard to ontology, Haag argued that a perfect representation of those ostensible objects of the world offered by common sense and by previous physics is not possible: The realist picture he offers via Local Quantum Physics is to be understood as a good, but only approximate characterization through science of reality itself because of inherent limitations corresponding to quantum coherence and quantum correlation. The ontology of LQP is one of events—probabilistically caused by previous event(s) and realized with the progression of time according with a fundamental causal arrow—linked to each other by causes that themselves are reified upon the occurrence of these events as they appear; these two sorts of ‘elements of reality’ give rise thereby to a causal network upon which space-time itself depends, rather than appearing in some independently existing space-time, and becomes a growing, historical, and present universe. Previously recognized physical objects are considered real if they serve as such causal links between realized events, for example, particles in scattering experiments. However, their individuality is limited.

LQP is an active area of research in which some technical questions which it intentionally raised remain in need of more precise answers. The solution of these would better indicate the relationship between Haag’s ontology, that of QFT, and the empirical results of physics. In particular, whether an adequate derivation of space-time from primitive events can be provided remains an open question.
